# H_2_S Alleviates Salinity Stress in Cucumber by Maintaining the Na^+^/K^+^ Balance and Regulating H_2_S Metabolism and Oxidative Stress Response

**DOI:** 10.3389/fpls.2019.00678

**Published:** 2019-05-28

**Authors:** Jing-Long Jiang, Yun Tian, Li Li, Miao Yu, Ru-Ping Hou, Xu-Ming Ren

**Affiliations:** ^1^School of Biological Science and Engineering, Shaanxi University of Technology, Hanzhong, China; ^2^School of Chemical and Environmental Science, Shaanxi University of Technology, Hanzhong, China

**Keywords:** salt stress, hydrogen hulfide, *Cucumis sativus* L., lipid peroxidation, Na^+^/K^+^ balance, expression of *SKOR* gene

## Abstract

Salinity stress from soil or irrigation water can significantly limit the growth and development of plants. Emerging evidence suggests that hydrogen sulfide (H_2_S), as a versatile signal molecule, can ameliorate salt stress-induced adverse effects. However, the possible physiological mechanism underlying H_2_S-alleviated salt stress in cucumber remains unclear. Here, a pot experiment was conducted with an aim to examine the possible mechanism of H_2_S in enhancement of cucumber salt stress tolerance. The results showed that H_2_S ameliorated salt-induced growth inhibition and alleviated the reduction in photosynthetic attributes, chlorophyll fluorescence and stomatal parameters. Meanwhile H_2_S increased the endogenous H_2_S level concomitant with increased activities of D/L-cysteine desulfhydrase and β-cyanoalanine synthase and decreased activities of O-acetyl-L-serine(thiol)lyase under excess NaCl. Notably, H_2_S maintained Na^+^ and K^+^ homeostasis via regulation of the expression of *PM H^+^-ATPase*, *SOS1* and *SKOR* at the transcriptional level under excess NaCl. Moreover, H_2_S alleviated salt-induced oxidative stress as indicated by lowered lipid peroxidation and reactive oxygen species accumulation through an enhanced antioxidant system. Altogether, these results demonstrated that application of H_2_S could protect cucumber seedlings against salinity stress, likely by keeping the Na^+^/K^+^ balance, controlling the endogenous H_2_S level by regulating the H_2_S synthetic and decomposition enzymes, and preventing oxidative stress by enhancing the antioxidant system under salinity stress.

## Introduction

Salinization of soil is pervasive throughout the globe, which is gradually becoming an earnest threat to world agriculture, affecting approximately 20% of arable irrigated land and consequently leading to the loss of United States$ 27.5 billion per annum (Abdel Latef et al., 2019). Salt stress is a major environmental factor that leads to significant inhibition of plant growth and decrease of productivity. Salt stress can greatly inhibit plant growth by decreasing biomass production and reducing the P_n_ and T_r_ (Chen et al., 2016). Chlorophyll fluorescence is considered a tool for interpreting stress tolerance in plants. Measurement of the chlorophyll fluorescence from PSII, the primary reactions of photosynthesis, provides an assessment of plant stress (Diao et al., 2014). Under adverse conditions, F_o_ is an important indicator of damage to the PSII reaction center, while the F_v_/F_m_ and F_v_/F_o_ ratios reflect its maximum photo-energy conversion efficiency and its potential photochemical efficiency. The F_v_/F_m_ and Φ_PSII_ represent the primary and actual light energy conversion capacities, respectively. Any decreases in F_v_/F_m_ and Φ_PSII_ indicate decline in photochemical activity of PSII (Kumar and Parsad, 2015). In general, the F_v_/F_m_, Φ_PSII_ and qL parameters have been calculated to reflect photochemical quenching, while NPQ is a non-photochemical-quenching parameter (Zhang et al., 2009). The factor qP represents the reduced state of the primary electron acceptor QA (Kumar and Parsad, 2015). Under saline conditions, there is a general decrease in qP parameters (F_v_/F_m_ and F_o_) and in the ETR, but increases in qP, Φ_PSII_ and NPQ.

It is well known that salt stress results in the over-accumulation of Na^+^ in plants cells, which competitively inhibit the uptake of K^+^, thus leading to a deficiency of K^+^ (Zhu, 2003; Munns and Tester, 2008). Plants have evolved varying mechanisms to adapt to salinity, including reducing Na^+^ influx into the root, control of Na^+^ xylem loading, Na^+^ retrieval from the xylem, Na^+^ recirculation in the phloem, Na^+^ efflux from the root, intracellular compartmentation of Na^+^ into the vacuoles, and Na^+^ secretion from the leaf (Cui et al., 2011; Kronzucker and Britto, 2011). The best way to limit Na^+^ accumulation in plants would be to reduce Na^+^ influx into the root in the first place, leading to improved salt tolerance of crop plants. The SOS pathway is essential for salt stress tolerance and maintaining ion homeostasis in the cytoplasm (Zhu, 2002). For example, a plasma membrane Na^+^/H^+^ antiporter (*SOS1*) is able to remove excess Na^+^ from cells (Feki et al., 2014). The *PM H^+^-ATPase* also plays an important role in the regulation of ion homeostasis in the cytosol when plants encountered salt stress (Binzel, 1995). On the other hand, the prevention of Na^+^-caused K^+^ leakage is also critical for plant salt tolerance (Lai et al., 2014). K^+^ channels or transporters are responsible for the K^+^ transport into or out of root cells. Several reports showed that the outward-rectifying K^+^channel such as *SKOR* is involved in K^+^ efflux (Lai et al., 2014). The *SKOR* is expressed in the root stele and responsible for K^+^ release to the xylem (Gaymard et al., 1998).

Plants under salinity stress usually produce excessive amounts of ROS, such as O_2_^•-^, OH, ^1^O_2_, and H_2_O_2_ (Yin et al., 2016). And the excessive accumulation of ROS is potentially harmful to proteins, DNA and lipids, leading to impairment of membrane integrity, enzyme inhibition and chlorophyll degradation. Therefore, ROS scavenging is important for plant survival and growth under salinity stress conditions. To combat with excessive accumulation of ROS, plants have evolved a protective strategy by activating antioxidant system, which includes non-enzymatic antioxidants, such as acid ASA and GSH, and enzymatic antioxidants, such as SOD (EC1.15.1.1), CAT (EC 1.11.1.6), POD (EC 1.11.1.7), GPX (EC 1.11.1.9), APX (EC 1.11.1.11), and GR (EC 1.6.4.2). The functions of ASA and GSH are closely related to their redox states (Kocsy et al., 2001) and plants can adjust redox states of ASA and GSH by modulating their regeneration and biosynthesis.

For a long time, H_2_S has been considered as merely a toxic by product of cell metabolism, but nowadays it is emerging as a novel gaseous signal molecule, which participates in seed germination, plant growth and development, as well as the acquisition of stress tolerance including cross-adaptation in plants (Li et al., 2016). H_2_S homeostasis is closely regulated by L-CD, EC 4.4.1.1, D-CD, EC 4.4.1.15, CAS, EC 4.4.1.9, OAS-TL, EC 2.5.1.47, and so on (Li et al., 2016). L-CD and D-CD can catalyze the degradation of L-/D-cysteine to produce H_2_S, amine and pyruvate. Additionally, H_2_S can be released from cysteine in the presence of hydrogen cyanide by CAS. On the other hand, OAS-TL can incorporate H_2_S into O-acetyl-L-serine to form cysteine, and its reverse reaction can release H_2_S. Generally, plants synthesize H_2_S via L-CD or D-CD, which respond to environment stress to promote stress tolerance.

Evidence in some plant species has proven that H_2_S participates in plant responses to salt stresses. Wang et al. (2012) reported that H_2_S can enhance *Medicago sativa* tolerance against salinity via the NO pathway, which is also confirmed by Chen et al. (2015) in barley seedling. It is reported that H_2_S can coordinate regulation of the SOS pathway to overcome the deleterious effects of salt stress in strawberry (Christou et al., 2013). Deng et al. (2016) also confirmed that H_2_S kept a lower Na^+^ concentration via the regulation the SOS1 pathway to alleviate growth inhibition in wheat seedlings under NaCl stress. Subsequently, Lai et al. (2014) reported that endogenous H_2_S can enhance salt tolerance by the establishment of redox homeostasis and preventing salt-induced K^+^ loss in seedlings of *Medicago sativa.* In addition, H_2_S can also synergistically regulate Na^+^/K^+^ balance, mineral homeostasis and oxidative metabolism in rice under excessive salt stress (Mostofa et al., 2015b). But these studies are commonly involved in one aspect, either Na^+^/K^+^ balance or antioxidation system, either in roots or leaves, and there are limited reports on the overall changes in morphology, photosynthesis, stomatal responses, ROS accumulation and the correlation between changes in the leaves and roots following exogenous H_2_S treatment. Furthermore, the underlying mechanisms through which H_2_S regulates salt tolerance, especially the changes in H_2_S homeostasis, are still elusive, requiring in-depth analysis at the physiological and biochemical levels. Cucumber (*Cucumis sativus* L.) is an economically important crop as well as a model plant for systematic investigations on many aspects.

In this study, a pot experiment was conducted with an aim to evaluate the possible mechanism by which H_2_S enhances tolerance of salinity stress in the important vegetable crop cucumber, with particular emphasis on maintenance of the Na^+^/K^+^ balance, and regulating of H_2_S metabolism and oxidative stress response.

## Materials and Methods

### Plant Culture and Treatments

Under salt stress, the morphological characters and physiological indexes of sensitive cultivars are more obvious than tolerant cultivars. When the sensitive plants were treated with H_2_S, the ameliorative effects of H_2_S will be easy to judge. Therefore, the sensitive cucumber (*C. sativus* L.) cultivar “Chunxiaqiuwang” was used in our experiments. Uniform seeds were sterilized in 10 % (v/v) sodium hypochlorite solution for 5 min followed by washing three times with distilled water. Sterilized seeds were germinated on moist filter paper in petri dishes at 25°C in the dark. After germination, seedlings were transplanted into perlite-filled plastic pots (240 mL, 65 × 45 × 70 mm, a seedling per pot) and watered with Hoagland’s solution. 10 mL Hoagland’s solution was applied for every other day after germination, which contained 5 mM KNO_3_, 5 mM Ca(NO_3_)_2_, 1 mM NH_4_H_2_PO_4_, 2 mM MgSO_4_, 10 μM MnSO_4_, 50 μM H_3_BO_3_, 0.7 μM ZnSO_4_, 0.2 μM CuSO_4_, 0.01 μM (NH_4_)_6_Mo_7_O_24_ and 70 μM Fe-EDTA-Na_2_. The seedlings of cucumber were maintained in a controlled growth chamber with a light/dark regime of 14/10 h, relative humidity of 70%, temperature of 25°C and a PAR of 800 μmol.m^-2^.s^-1^. When the first true leaf emerged, uniform and healthy seedlings were selected and divided into three groups for the following treatments. (i) Hoagland’s nutrient solution (as Control); (ii) Hoagland’s nutrient solution + 200 mM NaCl (as NaCl); (iii) Hoagland’s nutrient solution + 200 mM NaCl + 5 or 10, or 15 or 20 μM NaHS (as NaCl + NaHS). In our preliminary experiment, the phenotypic characteristics of plants with treated by H_2_S alone were no significant difference compared with the control group (data not shown). Thus there is no only H_2_S treatment group in our study. NaHS is commonly used as an H_2_S donor since it dissociates to water produce HS^-^ and Na^+,^ and then combination of HS^-^ with H^+^ produces H_2_S (Lin et al., 2012). NaHS is responsible for induction of stress tolerance, but not Na_2_S, Na_2_SO_4_, NaHSO_4_, Na_2_SO_3_, NaHSO_3,_ or CH_3_COONa (Deng et al., 2016). Each treatment was replicated three times under the same experimental conditions. The NaHS was purchased from Sigma (St Louis, MO, United States) and used as H_2_S donor. The remaining treatment solution, which was not fully absorbed by plants, was removed and 10 mL fresh treatment solution was irrigated every other day at 9:00 AM during the whole culture process. After 7 days of treatment, the parameters of photosynthesis and chlorophyll fluorescence were determine using the first true leaf of seedlings with different treatments. And finally, at least 24 seedlings per treatment group were harvested and weighed separately to determine various physiological and biochemical parameters, while other samples were immediately frozen in liquid N_2_ and then stored in a -80°C freezer until use.

### Morphological Assessment

Root length, seedling length (including aboveground and underground parts) and leaf area index were determined after 7 days of treatment. Leaf area index was measured using graph paper according to the method of Gupta et al. (2017). Plant roots were gently removed from the perlite, and then washed three times with deionized water to remove adhered perlite particles. After absorbing moisture from the root surface with a clean filter paper, the fresh weights of root and shoot were recorded, respectively. Shoot dry weight and root dry weight were, respectively, recorded after drying the same plants in an oven at 60 ° C until the weight became constant. Survival rate (%) was calculated as the number of survival seedlings / number of total seedlings. The rate of second leaf expansion (%) was calculated as the number of seedlings with appearance of the second leaf / number of total seedlings.

### Measurements of Photosynthesis, Chlorophyll Fluorescence, and Chlorophyll Content

The first true leaf was chosen to determine the parameters of photosynthesis, chlorophyll fluorescence and chlorophyll Content. And the P_n_ and T_r_ were measured using an LI-6400 portable photosynthesis system (LI-COR, Lincoln, NE, United States). The leaf chlorophyll fluorescence parameters were measured using a portable chlorophyll fluorometer (OS-30p+, Opti-Science, Inc., Tyngsboro, MA, United States). The F_o_ emission was determined on dark-adapted leaves. The F_m_ was obtained during a subsequent saturating light pulse. A second saturating pulse of white light was imposed to determine the F_m′_. The F_o′_ was determined by illuminating the leaf with far-red light for 3 s. The fluorescence parameters were calculated according to Hu and Yu (2014): maximal quantum yield of PSII photochemistry, F_v_/F_m_ = (F_m_-F_o_)/F_m_; effective quantum-use efficiency of PSII in light-adapted state, F_v′_/F_m′_ = (F_m′_-F_o′_)/F_m′_; quantum yield of PSII photochemistry, Φ_PSII_ = (F_m′_-F_t_)/F_m′_; qP = (F_m′_-F_t_)/(F_m′_-F_o′_); and NPQ = (F_m_-F_m′_)/F_m′_. The contents of Chlorophyll a (C_a_), chlorophyll b (C_b_), and C_x_+_c_ were measured on fresh leaves as described by Lichtenthaler and Wellburn (1983).

### Analysis of Stomatal Parameters by Scanning Electron Microscope

The first true leaves were selected to measure the stomatal opening by scanning electron microscopy (S-3400N; Hitachi, Tokyo, Japan). The leaf epidermis and stomata were observed and photographed under 15 kv. The diameter of the stomata was measured by Motic Images Advanced 3.2 (Notic China Group Co., Ltd., China) software at 300× magnification.

### Analysis of Electrolyte Leakage and Malondialdehyde Content

Membrane integrity was evaluated in the leaves and roots by measuring electrolyte leakage. Fresh leaf or root samples (50 mg) were cut in small pieces followed by repeated washings with double distilled water. And then the samples were incubated at 28°C for 2 h in test tubes containing 10 mL of double distilled water for determining EC1 using a conductivity meter (Lei-Ci, DDSJ-308F, Shanghai, China). The same samples were again kept in water bath at 100°C for 20 min and the EC2 was recorded at room temperature. The electrolyte leakage was calculated as electrolyte leakage (%) = (EC1/EC2) × 100%. MDA content in the leaves or roots was measured by TBA reaction according to Shi et al. (2014) with modifications. Leaves or roots tissue (0.5 g) was homogenized in 10 mL of 0.1% (w/v) TCA, and the extract was centrifuged at 5, 000 × *g* for 10 min at 4°C. To measure MDA, 1.5 mL of the supernatant was added into 1.5 mL of 0.5% (w/v) TBA made in 5 % TCA. The mixture was heated at 100°C for 20 min and then quickly cooled in an ice bath. After centrifuging at 7, 888 × *g* for 10 min, the absorbance of the supernatant was measured at 450 nm, 532 nm, and 600 nm, respectively. The MDA content of leaves or roots (per 0.5 g, FW) was calculated using the formula: C (nmol. g^-1^ FW) = 20 × [6.45 (OD_532_ – OD_600_) – 0.56 OD_450_].

### Determination of Endogenous O_2_^•-^ and H_2_O_2_ Levels

The production of O_2_^•-^ was determined using the protocol described by Jiao et al. (2011) with some modifications. Samples (0.5 g) were homogenized in an ice bath with 1.2 mL of phosphate buffer (pH 7.8) and centrifuged at 5, 000 × *g* for 10 min at 4°C. The supernatant was reacted with 1 mL of hydroxylamine hydrochlorides for 1 h, then 1 mL of p-aminobenzene sulfonic acid and 1 ml of α-naphthylamine were added. The solution was kept at 25°C for 20 min. The optical density value of the solution was measured with a spectrophotometer at 530 nm using NaNO_2_ as the standard curve and the corresponding calibration curves was Y = 552.16 X + 4.365 (*R*^2^ = 0.9903) H_2_O_2_ was measured spectrophotometrically according to Jiang et al. (2012). Roots and leaves (0.2 g) were homogenized in an ice bath with 1 mL of 0.1% TCA and centrifuged at 12, 000 × *g* for 20 min at 4°C. The supernatant was used for measuring H_2_O_2_ content. The reaction mixture consisted of 0.5 mL of the extracted supernatant, 0.5 mL of 10 mM potassium phosphate buffer (pH 7.0) and 1 mL of 1 M potassium iodide. The reaction was developed for 1 h in darkness before the absorbance was measured at 390 nm. The blank was 0.1% TCA in the absence of root extract. The amount of H_2_O_2_ was calculated using a standard curve prepared with known concentrations of H_2_O_2_. The corresponding calibration curves was Y = 82.69 X – 1.11 (*R*^2^ = 0.9959).

### Determination of Endogenous H_2_S Level and the Activities of L-CD, D-CD, CAS, and OAS-TL

Endogenous H_2_S content was determined by the formation of methylene blue from dimethyl-p-phenylenediamine in H_2_SO_4_ according to the method described previously (Zhang et al., 2008). Samples (0.5 g) were extracted in 1 mL phosphate buffer solution (50 mM, pH 6.8) containing 0.2 M ASA and 0.1 M EDTA. Subsequently, 1 M HCl (0.5 mL) was added to the homogenate in a test tube for releasing H_2_S, and 0.5 mL of 1% (w/v) zinc acetate was used to absorb H_2_S. After 30 min reaction, 0.3 mL 5 mM N,N-dimethyl-p-phenylenediamine dihydrochloride dissolved in 3.5 mM H_2_SO_4_ was injected into the trap. Then 0.3 mL of 50 mM ferric ammonium sulfate in 100 mM H_2_SO_4_ was injected into the trap. The amount of H_2_S in zinc acetate traps was determined spectrophotometrically at 670 nm, after leaving the mixture for 15 min at room temperature. Solutions with different concentrations of Na_2_S were prepared, treated in the same way as the assay samples and were used for the quantification of H_2_S.

Samples (0.3 g) were ground with a mortar and pestle in liquid nitrogen and soluble proteins were extracted with 1.5 mL of cold extraction buffer containing 20 mM Tris–HCl (pH 8.0), 0.1% (w/v) DTT and 0.2% (w/v) sodium ascorbate. The homogenate was centrifuged at 13, 000 × *g* at 4°C for 15 min. The resulting supernatant was used for the determination of the activities of L/D-CD, CAS, and OAS-TL. Total L-CD activity was determined by the release of H_2_S from L-cysteine as described in Riemenschneider et al. (2005) with minor modification. The assay contained in a total volume of 1 mL: 0.1 mL of 10 mM L-cysteine, 0.8 mL 100 mM Tris–HCl (containing 2.5 mM DTT, pH 9.0) and 0.1 mL of protein solution. After incubation for 60 min at 30° C, the reaction was terminated by adding 0.1 mL of 30 mM FeCl_3_ dissolved in 1.2 N HCl and 0.1 mL of 20 mM N, N-dimethyl-p-phenylenedi-amine dihydrochloride dissolved in 7.2 N HCl. The formation of methylene blue was determined at 670 nm, and known concentrations of Na_2_S were used in the calibration curve. D-CD activity was determined in the same way as L-CD activity except with D-cysteine instead of L-cysteine and the pH of the Tris–HCl buffer was 8.0. One unit of enzyme activity is defined as the amount of enzyme that produces 1.0 mmol/min H_2_S under the stated assay conditions and expressed as U g^-1^ FW. The total OAS-TL activity was determined following the method described Bloem et al. (2004). CAS activity was measured by the release of H_2_S from L-cysteine in the same way as L-CD activity with the following modifications: the 1.0 mL volume of reaction mixture contained 0.1 mL of 10 mM L-cysteine, 0.7 mL 100 mM Tris–HCl (pH 9.0), 0.1 mL of 7.5 mM KCN and 0.1 mL of protein solution, as described in Meyer et al. (2003).

### Determination of Na^+^ and K^+^ Content and Na^+^/K^+^

Dried plant material (200 mg) was subjected to acid digestion in 10 mL digestion mixture [HClO_4_: HNO_3_: H_2_SO_4_ (5: 1: 1, v/v)] on a hot plate (120°C) until the volume was reduced to 1 mL. After diluting with double distilled water, the Na^+^ and K^+^ contents were estimated using the flame photometer (Horneck and Hanson, 1998).

### Extraction of Total RNA and Real-Time RT-PCR Analysis

To study the effect of NaHS treatment on the expression of the *SOS1*, *SKOR*, and *PM H^+^-ATPase* genes in cucumber leaves and roots in response to salt stress, 18-day-old seedlings treated with either Hoagland’s nutrient solutions (control group), 200 mM NaCl, or 200 mM NaCl + 15.0 μM NaHS for a week were harvested. Total RNA of cucumber leaves or roots (100 mg) was isolated by grinding with mortar and pestle in liquid nitrogen to a fine powder and using TriZol Reagent (Invitrogen) in accordance with the manufacturer’s protocol. The total RNA samples were treated with RNAase-free DNase (TaKaRa Bio Inc., Dalian, China) to eliminate traces of DNA, followed by quantification using the NanoDrop 2000 (Thermo Fisher Scientific, Wilmington, DE, United States). Total RNA (2 μg) was reverse-transcribed using an oligo d (T) primer (50 μM, 1 μL) and M-MLV reverse transcriptase (200 U/μL, 1 μL) (BioTeke, Beijing, China). Real-time quantitative RT-PCR reactions were performed using an ABI 7000 (Applied Biosystems) with SYBR^®^Premix ExTaq^TM^ (TaKaRa Bio Inc., China) and the cycling conditions of denaturation at 95°C for 5 min, followed by 40 cycles of denaturation at 95°C for 15 s, annealing at 60°C for 30 s, and extension at 72°C for 15 s. Using specific primers (Supporting information, Supplementary Table S1), the expression levels of the genes were presented as values relative to the corresponding control samples under the indicated conditions, with normalization of data to the geometric average of internal control gene *GAPDH* (a housekeeping genes). Three independent replicates were performed for each sample. The comparative threshold cycle (Ct) method was used to determine the relative amount of gene expression. Relative gene expression levels were calculated via the 2^-ΔΔCT^ method. The relative gene expression was determined as previously described by Livak and Schmittgen (2001).

### Measurements of ASA, GSH, SOD, CAT, GR, and POD

The ASA content was determined using the DCIP method. Samples (0.50 g) were homogenized in 3.0 mL of 2 % oxalic acid supplemented with 0.50 mL of 30 % ZnSO_4_ and 0.50 mL of 15% K_4_Fe(CN)_6_⋅3H_2_O. The volume of the extract was adjusted to 10.0 mL with 1% oxalic acid, followed by centrifugation at 10, 000 × *g* for 10 min. Then 3 mL of the supernatant was mixed with 2.0 mL of dye solution (0.25% DCIP and 0.20% NaHCO_3_) and 3 mL of dimethylbenzene. The absorbance was read at 500 nm, and the content of ASA was calculated using a standard curve. Total GSH was estimated using a kit (Jiancheng Bioengineering Institute, Nanjing, China). The absorbance of GSH was measured at 420 nm according to manufacturer’s instructions. CAT activity was measured according to the methods described by Velikova et al. (2000) using 10 mM potassium buffer (pH 7.00), with 100 μL of enzyme extract, and 33 mM H_2_O_2_. H_2_O_2_ was assayed from the decrease in absorbance in optical density at 240 nm, and the activity was calculated using the extinction coefficient of 40 mM cm^-1^ of H_2_O_2_. GR activity was determined by measuring the absorbance change at 340 nm due to oxidation of NADPH (Foyer and Halliwell, 1976). SOD and POD activities were assayed as described by Chen et al. (2013).

### Statistical Analysis

Data were analyzed by one-way ANOVA using SPSS (version 21.0.0; IBM, Armonk, NY, United States). Different letters on the graphs or in the tables indicated that the mean values were statistically different at the *P* < 0.05 level.

## Results

### H_2_S Alleviates NaCl-Induced Inhibition of Cucumber Plant Growth

**FIGURE 1:**
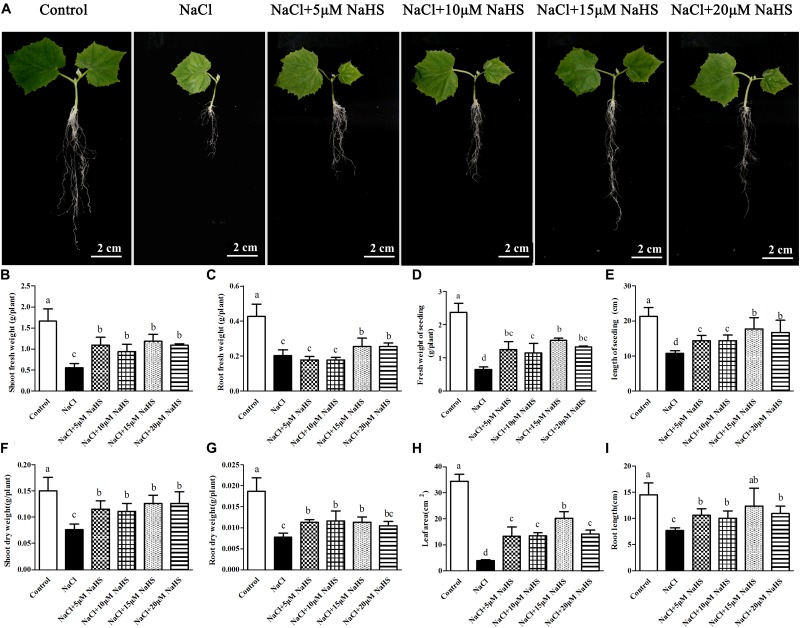
Effects of NaHS treatment on the NaCl-induced changes in cucumber seedling growth **(A)**. Plant growth was evaluated by the indexes of shoot fresh weight **(B)**, root fresh weight **(C)**, fresh weight of seedling **(D)**, length of seedling **(E)**, shoot dry weight **(F)**, root dry weight **(G)**, leaf area **(H)** and root length **(I)** in *Cucumis sativus* L. cv. Chunxiaqiuwang grown under 200 mM NaCl. Each value is the mean of three biological replicates, and the vertical bars represent the standard errors. Values sharing the same lower case letters are insignificant as per Duncan’s test at *P* < 0.05.

The morphology, survival rate, and expansion rate of the second leaf were determined after cucumber seedlings exposed to 200 mM NaCl for 7 days. Under salt stress, most seedlings began to show leaf wilting, lodging and inhibition of the second leaves compared with control group (Supporting information, Supplementary Figure S1). In order to analyze the alleviation effect of NaHS on growth inhibition induced by NaCl in a dose-dependent manner, series of concentrations of NaHS were added to the nutrient solution. In cucumber seedlings, 15 or 20 μM NaHS significantly alleviated the inhibition of plant growth during salt stress (Supplementary Figure S1A). Salinity treatment significantly (*P* < 0.05) reduced the survival rate by 89 %, the rate of expansion of the second leaf by 62 % (Supplementary Figures S1B,C) and significantly (*P* < 0.05) affected general growth (Figure 1A). Compared with the control, the shoot fresh weight, root fresh weight, seedling fresh weight, seedling total length, shoot dry weight, root dry weight, leaf area and root length were significantly (*P* < 0.05) reduced by 66%, 58%, 65%, 43%, 50%, 53%, 90%, and 48% under salt stress, respectively (Figure 1B–I). However, all growth parameters showed different degree ameliorating effects with application of different concentrations of NaHS (Figure 1 and Supplementary Figure S1). For example, the combination NaCl+15 μM NaHS significantly (*P* < 0.05) reduced shoot fresh weight, root fresh weight, seedling fresh weight, seedling total length, shoot dry weight, root dry weight, leaf area and root length by 28%, 40%, 31%, 14%, 17%, 42%, 41%, and 15%, respectively, as compared to the control (Figure 1B–I). Thus, these results indicated that exogenous supplementation with 15 μM NaHS was the most ameliorating effect in boosting cucumber tolerance to NaCl stress.

### Effects of H_2_S on Photosynthesis Under Excess NaCl

In comparison with the control, a significant (*P* < 0.05) decline in the P_n_ (by 14.3%) and a sharp decline in the T_r_ (by 94%) were recorded in the leaves of salt-stressed cucumber plants (Table 1). The combination of NaCl and 15 μM NaHS showed a decline in the P_n_ (by 2.4%) and T_r_ (by 90.4%) compared with the control, respectively (Table 1). Nevertheless, 5 μM NaHS did not significantly affect the Tr under salt stress. In addition, 20 μM NaHS alleviated the declines of P_n_, but did not cause a significant change of T_r_ under salt stress.

**Table 1:** Effects of NaHS treatment on the P_n_, T_r_, F_0_, F_v_/F_m_, F_v_/F_0_, Φ_PSII_, NPQ, and qP values and the contents of C_a_, C_b_, C_a+b_, and C_x+c_ in *Cucumis sativus* L. cv. Chunxiaqiuwang grown under 200 mM NaCl.

Index	Control	NaCl	NaCl+5 μM NaHS	NaCl+10 μM NaHS	NaCl+15 μM NaHS	NaCl+20 μM NaHS
P_n_ (μmol. m^-2^s^-1^)	8.037 ± 0.286a	6.888 ± 0.095d	7.427 ± 0.129c	7.678 ± 0.113b	7.848 ± 0.119ab	7.443 ± 0.090c
T_r_ (mmol. m^-2^s^-1^)	5.236 ± 0.205a	0.291 ± 0.006c	0.327 ± 0.028c	0.371 ± 0.023bc	0.502 ± 0.019b	0.427 ± 0.037bc
F_0_	183.00 ± 8.08d	253.50 ± 3.70a	211.50 ± 5.74bc	217.75 ± 9.78b	204.25 ± 1.71c	214.00 ± 6.27b
F_v_/F_m_	0.801 ± 0.005a	0.753 ± 0.009c	0.775 ± 0.019b	0.771 ± 0.009b	0.797 ± 0.001a	0.781 ± 0.008b
F_v_/F_0_	4.097 ± 0.096a	3.085 ± 0.136d	3.761 ± 0.069bc	3.642 ± 0.306c	3.914 ± 0.033ab	3.658 ± 0.085c
Φ_PSII_	0.735 ± 0.004a	0.685 ± 0.002c	0.716 ± 0.004b	0.710 ± 0.013b	0.720 ± 0.004b	0.718 ± 0.000b
NPQ	0.175 ± 0.015d	0.312 ± 0.007a	0.292 ± 0.027ab	0.288 ± 0.015ab	0.266 ± 0.007bc	0.242 ± 0.039c
qP	3.614 ± 0.076a	3.173 ± 0.020b	3.439 ± 0.018ab	3.496 ± 0.226a	3.503 ± 0.024a	3.478 ± 0.060a
C_a_ (mg. g^-1^ FW)	1.315 ± 0.120a	0.812 ± 0.009d	0.841 ± 0.005d	0.924 ± 0.062cd	1.168 ± 0.082b	1.005 ± 0.016c
C_b_ (mg. g^-1^ FW)	0.356 ± 0.039a	0.224 ± 0.006c	0.235 ± 0.001c	0.253 ± 0.017c	0.325 ± 0.017ab	0.299 ± 0.017bc
C_a+b_ (mg. g^-1^ FW)	1.620 ± 0.160a	1.036 ± 0.015d	1.074 ± 0.009d	1.177 ± 0.079cd	1.493 ± 0.099ab	1.296 ± 0.003bc
C_x+c_ (mg. g^-1^ FW)	0.307 ± 0.023a	0.199 ± 0.003d	0.206 ± 0.000d	0.220 ± 0.013cd	0.261 ± 0.022b	0.244 ± 0.019bc


*Each value is the mean of three biological replicates. Values sharing the same lower case letters are insignificant as per Duncan’s test at *P* < 0.05.*

As shown in Table 1, salt stress significantly (*P* < 0.05) reduced F_v_/F_m_ (by 6%), F_v_/F_o_ (by 25%),Φ_PSII_ (by 7%) and qP (by 12%) compared with the control, while 15 μM NaHS treatment reduced these by 0.5%, 4.5%, 2% and 3.1%, respectively, compared with the control. In contrast, comparing with the control, the parameters of F_o_ and NPQ significantly (*P* < 0.05) increased by 39% and 78% under salt stress. As expected, 15 μM NaHS significantly reversed the increase in F_o_ (by 10.4%) and NPQ (by 34.2%) under salt stress (Table 1).

Salt stress resulted in a decrease in the contents of the photosynthetic pigments C_a_, C_b_, C_a+b_ and C_x+c_ compared to the non-treated plants. Compared with the control, salt stress reduced C_a_, C_b_, C_a+b_ and C_x+c_ content by 38%, 37%, 36%, and 35%, respectively, while 15 μM NaHS treatment reduced C_a_, C_b_, C_a+b_, and C_x+c_ content by 11.2%, 8.7%, 7.8% and 15% (Table 1).

### Effects of H_2_S on the Stomatal Index Under Excess NaCl Conditions

**FIGURE 2:**
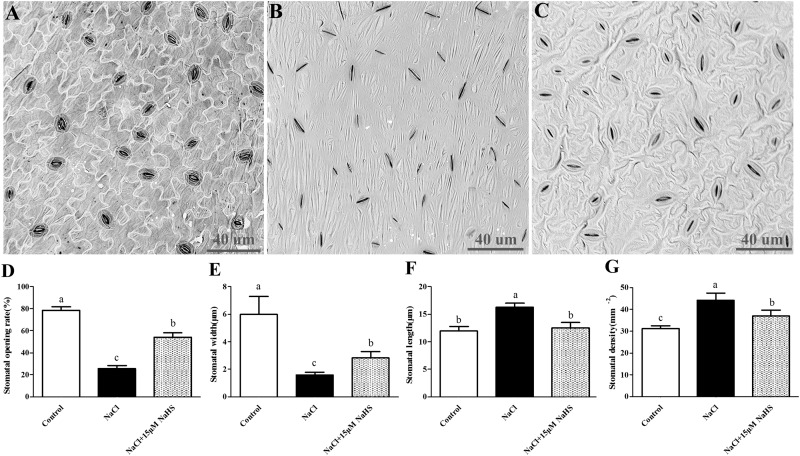
Scanning electron micrographs of abaxial leaf surfaces of cucumber showing the numbers and opening of stoma in control group **(A)**, 200 mM NaCl treatment group **(B)** and the 15 μM NaHS + 200 mM NaCl treatment group **(C)**. Effects of NaHS treatment on the changes of stomatal opening rate **(D),** stomatal width **(E)**, stomatal length **(F)**, and stomatal density **(G)** of *Cucumis sativus* L. cv. Chunxiaqiuwang under different treatments. Each value is the mean of three biological replicates, and the vertical bars represent the standard errors. Values sharing the same lower case letters are insignificant as per Duncan’s test at *P* < 0.05.

To determine the effects of H_2_S on stomata in cucumber, the abaxial leaf surface was observed by a scanning electron microscope to analyze several stomatal indexes (Figure 2A–C). Salt stress induced stomatal closure and resulted in abnormal morphology of the epidermal and guard cells compared to the untreated control plants, whereas application of 15 μM NaHS significantly reversed these damages. Salt stress caused a noticeable reduction in stomatal opening rate by 67% (Figure 2D) and stomatal width by 74% (Figure 2E), and a significant increase in stomatal density by 41% (Figure 2F) and stomatal length by 36% (Figure 2G) compared with control plants. However, 15 μM NaHS treatment reduced stomatal opening rate by 30.8% (Figure 2D) and stomatal width by 52.8% (Figure 2E), and increased stomatal density by 12.6% (Figure 2F) and stomatal length by 12% (Figure 2G) compared with control plants. The diverse responses of the stomata indicated that there is co-ordination of the physiological (i.e., aperture) and morphological (i.e., stomatal density) changes induced by H_2_S that control gas exchange and Tr when encountering unfavorable environments.

### Effects of H_2_S Treatment on Electrolytic Leakage, Lipid Peroxidation, and ROS Accumulation Under Excess NaCl

**FIGURE 3:**
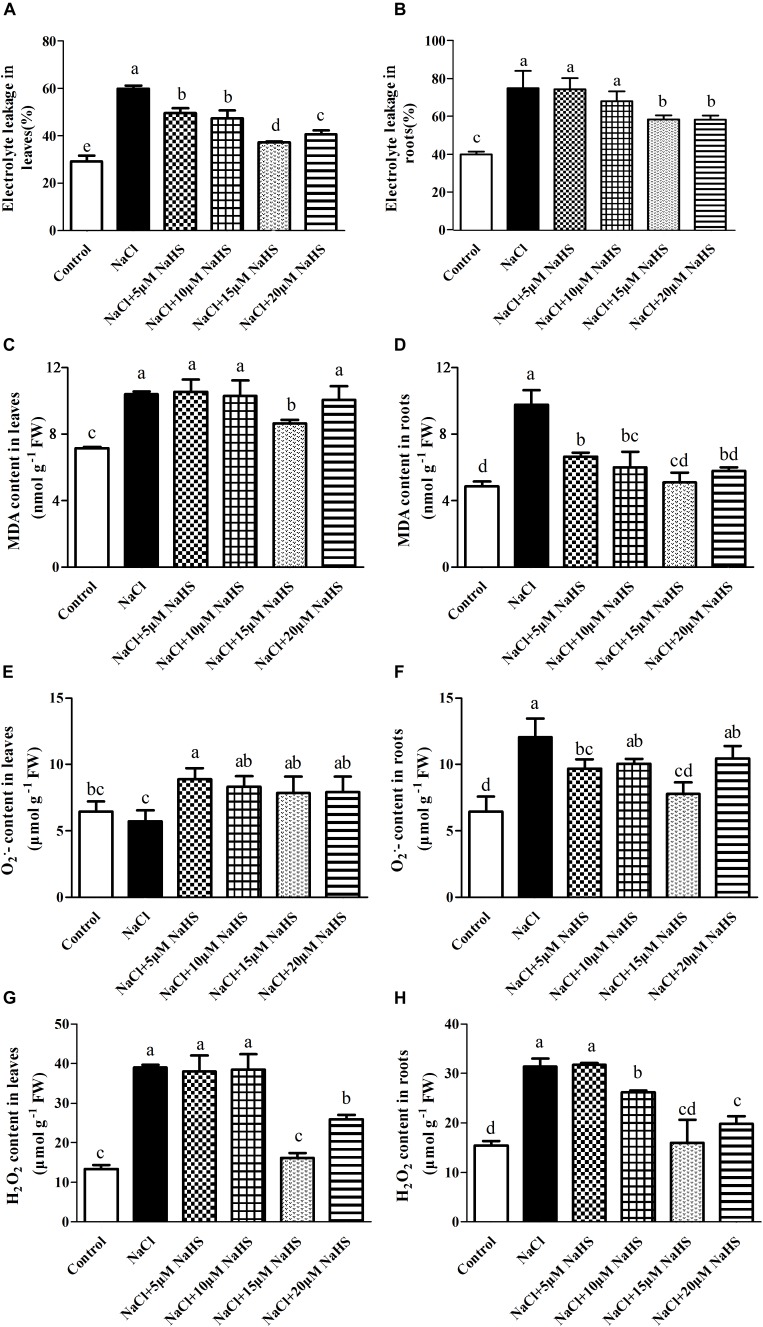
Effects of NaHS treatment on the NaCl-induced changes of electrolyte leakage in leaf **(A)** and root **(B)**, MDA content in leaf **(C)** and root **(D)**, O_2_^•-^ in leaf **(E)** and root **(F)**, and H_2_O_2_ content in leaf **(G)** and root **(H)** of *Cucumis sativus* L. cv. Chunxiaqiuwang. Each value is the mean of three biological replicates, and the vertical bars represent the standard errors. Values sharing the same lower case letters are insignificant as per Duncan’s test at *P* < 0.05.

The exposure of the cucumber plants to salinity significantly (*P* < 0.05) increased electrolyte leakage by 1.1 fold in leaves (Figure 3A) and by 87.2% in roots (Figure 3B), and increased the MDA content by 45% in leaves (Figure 3C) and by 1.0 fold in roots (Figure 3D) compared with the control. However, treatment of salt-stressed plants with 15 μM NaHS resulted in an increase in electrolyte leakage by 27.6% in leaves and by 46% in roots (Figure 3A,B) and an increase in the MDA content by 21% in leaves and by 5.4% in roots as compared to the control (Figure 3C,D). These results indicated that NaHS clearly reduces cell membrane injury.

Salt stress treatment did not change the O_2_^•-^ accumulation in cucumber leaves (Figure 3E), but it did increase by nearly 2.1 fold in roots as compared to control plants (Figure 3F). While different concentration of NaHS treatment increased the accumulation of O_2_^•-^ in cucumber leaves and roots as compared to the control (Figure 3E,F). Salt stress caused a dramatic increase in H_2_O_2_ by 1.92 fold in leaves (Figure 3G) and 1.04 fold in roots (Figure 3H) compared with the control. Treatment with 15 μM NaHS increased the H_2_O_2_ by 21.7% in leaves (Figure 3G) and 19.7% in roots compared with the control (Figure 3H). These results indicated that exogenous application of NaHS suppresses ROS accumulation, thereby protecting cucumber plants from NaCl-induced oxidative stress.

### Effects of NaHS Treatment on Endogenous H_2_S Levels and H_2_S Metabolism Under Excess NaCl

**FIGURE 4:**
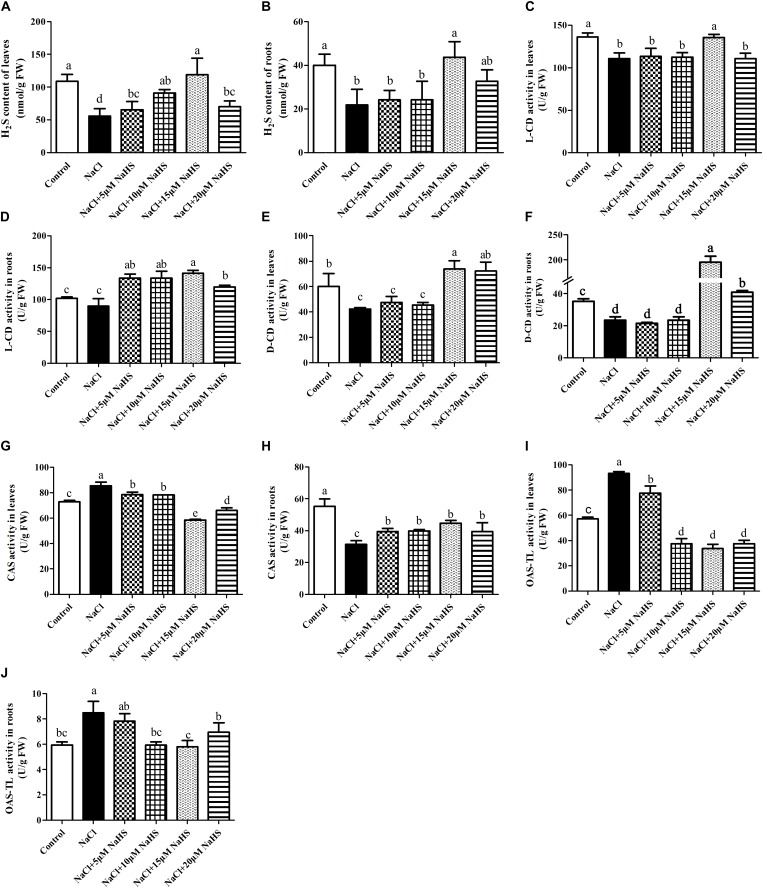
Effects of NaHS treatment on the NaCl-induced changes in endogenous H_2_S levels in leaf **(A)** and root **(B)** and activities of L-CD in leaf **(C)** and root **(D)**, D-CD in leaf **(E)** and root **(F)**, CAS in leaf **(G)** and root **(H)**, and OAS-TL in leaf **(I)** and root **(J)** of *Cucumis sativus* L. cv. Chunxiaqiuwang. Each value is the mean of three biological replicates, and the vertical bars represent the standard errors. Values sharing the same lower case letters are insignificant as per Duncan’s test at *P* < 0.05.

In comparison with the control, a sharp decline in the content of endogenous H_2_S was recorded in leaves (by 49%) (Figure 4A) and roots (by 45%) (Figure 4B) of salt-treated cucumber plants, whereas 15 μM NaHS treatment improved the content of endogenous H_2_S by 9.9% in leaves (Figure 4A) and by 9.1% in roots (Figure 4B) compared with the control. Compared with the control, salt stress significantly (*P* < 0.05) decreased the activity of L-CD by 19% in leaves (Figure 4C) and 12% in roots (Figure 4D). The same change was found in the activity of D-CD after salt stress, which reduced by 13% in leaves (Figure 4E) and 33 % in roots compared with the control (Figure 4F), As expected, these changes were reverted by different concentrations of NaHS treatment. For example, treatment with 15 μM NaHS significantly (*P* < 0.05) improved the activities of L-CD (38.7% in roots) and D-CD (by 52.7% in leaves and 4.6 fold in roots) compared with the control (Figure 4C–F). These results indicated that NaHS treatment significantly enhanced the activities of L-CD and D-CD in cucumber seedlings under salt stress.

As for the activity of CAS, a completely opposite trend was found between leaves and roots. Salt stress significantly (*P* < 0.05) improved the activity of CAS in leaves by 19% (Figure 4G) and reduced it by 43% in roots (Figure 4H) compared with the control. Application of 5 or 10 μM NaHS enhanced the activity of CAS by 9.4% or 8.9% in leaves, in contrast, 15 or 20 μM NaHS reduced it by 31.7% or 22.8% compared with the control. In the roots, 5∼20 μM NaHS all reduced the activity of CAS in different degree compared with the control group (Figure 4G,H). In addition, the OAS-TL activity significantly (*P* < 0.05) increased in both leaves (by 65%) (Figure 4I) and roots (43%) (Figure 4J) under salt stress. Similarly, these effects were reverted by different concentrations of NaHS as compared to the plants receiving salinity treatment alone. For example, 15 μM NaHS treatment reduced the activity of OAS-TL by 42% in leaves and 2.4% in roots as compared to the control (Figure 4I,J).

### Effects of H_2_S on Na^+^ and K^+^ Homeostasis Under Excess NaCl

**FIGURE 5:**
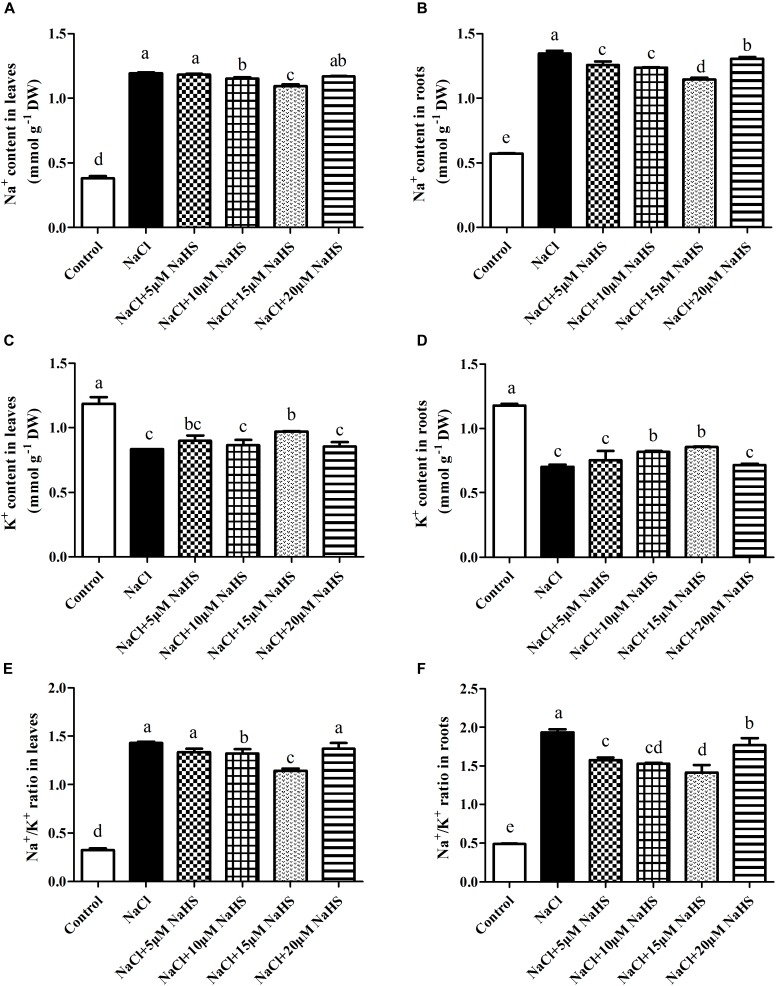
Effects of NaHS treatment on the NaCl-induced changes of Na^+^ content **(A,B)**, K^+^ content **(C,D)**, and Na^+^/K^+^ ratio **(E,F)** in leaf and root of *Cucumis sativus* L. cv. Chunxiaqiuwang grown under 200 mM NaCl. Each value is the mean of three biological replicates, and the vertical bars represent the standard errors. Values sharing the same lower case letters are insignificant as per Duncan’s test at *P* < 0.05.

In plants, maintaining a lower Na^+^/K^+^ ratio is critical to its ability to tolerate salt stress. Compared with the control, a sharp increase in the Na^+^ content was observed in leaves (by 2.13 fold) and roots (1.35 fold) under salt stress (Figure 5A,B). On the contrary, a significant decrease in the K^+^ content was recorded in leaves (by 30%) and roots (by 41%) under salt stress as compared to the control (Figure 5C,D). Accordingly, the Na^+^/K^+^ ratio significantly increased in leaves (by 3.4 fold) and roots (by 3 fold) of salt-treated plants than the control (Figure 5E,F). As expected, treatment with 15 μM NaHS improved the Na^+^ content in leaves by 1.9 fold and roots by 1.0 fold, and reduced the K^+^ content in leaves by 17.8% and roots by 28% compared with the control, indicating that 15 μM NaHS significantly reversed the increase of Na^+^ and the decrease of K^+^ under salt stress. In addition, the Na^+^/K^+^ ratio was significantly lower in the NaHS-treated plants when compared with the plants treated with salt alone (Figure 5E,F). Thus, H_2_S eases excessive Na^+^ uptake and maintains the Na^+^/K^+^ homeostasis at the cellular level in cucumber plants under salt stress.

### Effects of H_2_S on Expression of *PM H^+^-ATPase*, *SOS1*, and *SKOR* Under Excess NaCl

**FIGURE 6:**
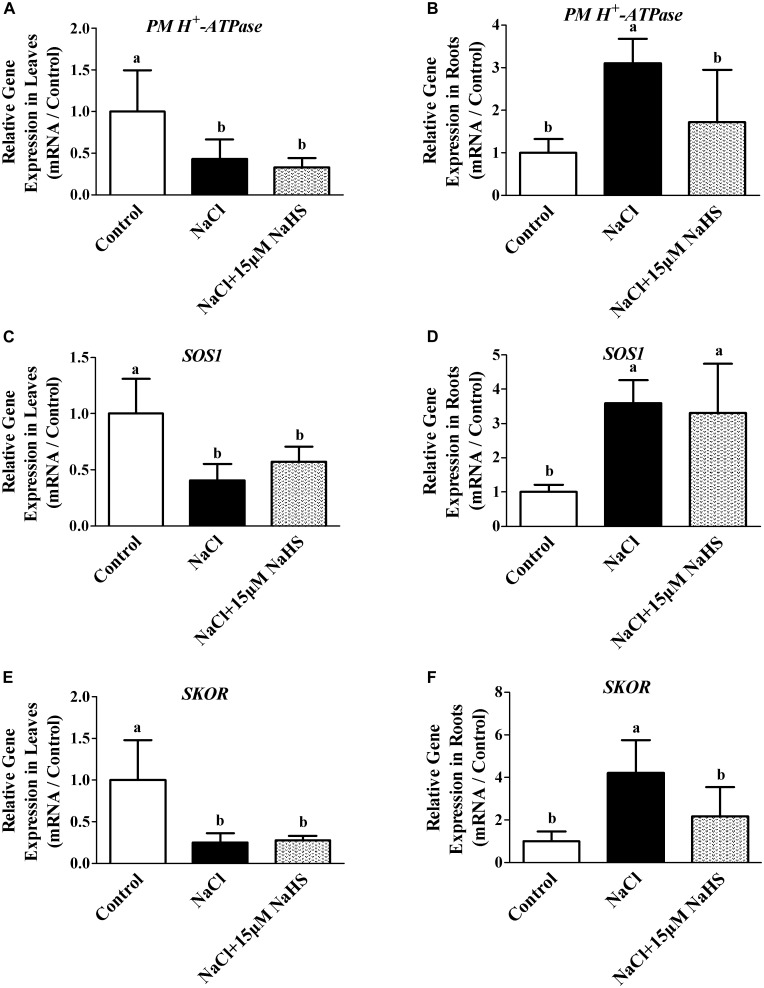
Effects of NaHS treatment on the NaCl-induced changes in expression of *PM H^+^-ATPase* in leaf **(A)** and root **(B)**, *SOS1* in leaf **(C)** and root **(D)**, and *SKOR* in leaf **(E)** and root **(F)** in *Cucumis sativus* L. cv. Chunxiaqiuwang grown under different treatments. Each value is the mean of three biological replicates, and the vertical bars represent the standard errors. Values sharing the same lower case letters are insignificant as per Duncan’s test at *P* < 0.05. Total RNAs were extracted from roots and leaves and subjected to reverse transcription followed by real-time PCR. *GAPDH* was used as an internal control. The transcript level in roots and leaves in the control group was defined as “1” for each gene, and other treated samples were expressed relative to the corresponding control. The columns labeled with different letters are significantly different at *P* < 0.05. Error bars are SE (*n* = 3).

To further characterize the effect of H_2_S on the modulation of Na^+^/K^+^ homeostasis in cucumber seedlings upon salt stress, the transcript levels of *PM H^+^-ATPase*, *SOS1* and *SKOR* were analyzed. Real-time RT-PCR analysis showed that NaCl treatment resulted in the significant decreases in the transcripts levels of *PM H^+^-ATPase* by 66.9% (Figure 6A), *SOS1* by 42.7% (Figure 6C) and *SKOR* by 72.4% (Figure 6E) in cucumber leaves compared with the controls, respectively. In contrast, the relative expression of *PM H^+^-ATPase*, *SOS1* and *SKOR* were significantly increased by 72.6% (Figure 6B), 2.3 fold (Figure 6D) and 1.17 fold (Figure 6F) in the roots under NaCl stress compared with the control. The NaCl-induced increases in *PM H^+^-ATPase*, *SOS1* and *SKOR* transcripts were significantly reversed by 15 μM NaHS treatments in the cucumber roots. As shown in Figure 6, compared with the control, treatment with 15 μM NaHS+NaCl reduced the relative expression of *PM H^+^-ATPase*, *SOS1* and *SKOR* by 57.1%, 59.5% and 75.1% in leaves, respectively. On the contrary, treatment with 15 μM NaHS+NaCl improved the relative expression of *PM H^+^-ATPase*, *SOS1* and *SKOR* by 2.1 fold, 2.59 fold and 3.21 fold in leaves, respectively, as compared to the control.

### Effects of H_2_S on the Antioxidant System

To evaluate the role of H_2_S in mediating salt-induced antioxidant system, the levels of non-enzymatic antioxidants (ASA and GSH) and the activities of antioxidant enzymes (SOD, CAT, GR, and POD) were analyzed in leaves and roots of cucumber plants. As show in Table 2, compared with the control, the contents of ASA and GSH and the activity of GR were significantly increased by 98%, 73%, and 61% in the leaves, but they were decreased by 88%, 23%, and 2.25 fold, in the roots under NaCl treatment. However, application of exogenous NaHS ameliorated the effect of salt stress. For example, 15 μM NaHS treatment significantly increased the ASA content by 42.6% and the GR activity by 9.1%, and decreased GSH by 44% in the leaves as compared to the control. On the contrary, 15 μM NaHS treatment decreased the ASA content by 23.9% and the GR activity by 27.8%, and increased GSH by 48.7% in the roots as compared to the control. Compared with the control, SOD activity was increased under salt stress in the leaves by 23% and roots by 43%. Application of NaHS showed a synergistic effect resulting in decreased SOD activity in the leaves and roots under NaCl stress. The application 15 μM NaHS significantly decreased SOD activity in the leaves by 19% and roots by 15% as compared to NaCl stress alone. The NaCl treatment significantly decreased the activity of CAT in the leaves by 6 % and roots by 59% as compared to control, but increased the POD activity in the leaves by 25% and roots by 10%. Application of NaHS reversed these changes in the leaves and roots induced by salt stress.

**Table 2:** Effects of NaHS treatment on ASA and GSH contents and the activities of SOD, CAT, GR, and POD in leaves and roots of *Cucumis sativus* L. cv. Chunxiaqiuwang grown under 200 mM NaCl stress.

Treatment	ASA (μg/mg)		GSH (μg/g)		SOD (U/mg protein)		CAT (U/mg protein)		GR (U/g protein)		POD (U/mg protein)	
	Leaves	Roots	Leaves	Roots	Leaves	Roots	Leaves	Roots	Leaves	Roots	Leaves	Roots
Control	2.09 ± 0.20c	1.88 ± 0.24a	10.63 ± 0.93c	3.94 ± 0.37c	2.49 ± 0.14b	2.02 ± 0.21c	0.36 ± 0.00a	0.05 ± 0.01cd	14.74 ± 2.32c	36.17 ± 5.68b	3.04 ± 0.27b	3.53 ± 0.03c
NaCl	4.13 ± 0.24a	0.22 ± 0.04c	18.37 ± 1.64a	3.02 ± 0.79d	3.07 ± 0.10a	2.89 ± 0.02a	0.34 ± 0.01b	0.02 ± 0.01e	52.25 ± 5.68a	14.07 ± 2.84b	3.81 ± 0.02a	3.88 ± 0.12ab
NaCl + 5 μM NaHS	3.59 ± 0.42ab	0.20 ± 0.07c	13.64 ± 1.11b	4.20 ± 0.37b	2.89 ± 0.01a	2.82 ± 0.05a	0.36 ± 0.01ab	0.04 ± 0.01de	46.22 ± 2.84a	18.09 ± 2.84ab	4.01 ± 0.16a	3.66 ± 0.06a
NaCl + 10 μM NaHS	3.36 ± 0.20ab	0.65 ± 0.17b	10.76 ± 0.73c	4.29 ± 0.15b	2.99 ± 0.23a	2.58 ± 0.11b	0.35 ± 0.01ab	0.07 ± 0.01bc	34.16 ± 2.84b	17.42 ± 6.14ab	4.10 ± 0.06a	3.94 ± 0.22a
NaCl + 15 μM NaHS	2.98 ± 0.22b	1.43 ± 0.31a	10.23 ± 0.37c	5.86 ± 0.30a	2.48 ± 0.09b	2.46 ± 0.02bc	0.37 ± 0.00a	0.08 ± 0.01a	16.08 ± 4.02c	26.13 ± 2.84a	3.45 ± 0.17b	3.54 ± 0.12c
NaCl + 20 μM NaHS	3.76 ± 0.97ab	0.46 ± 0.10bc	11.55 ± 1.11bc	4.46 ± 0.48b	2.53 ± 0.03b	2.85 ± 0.05a	0.36 ± 0.01a	0.08 ± 0.00ab	34.83 ± 6.14b	26.13 ± 8.53a	3.58 ± 0.036ab	3.67 ± 0.01bc


*Each value is the mean of three biological replicates. Values sharing the same lower case letters are insignificant as per Duncan’s test at *P* < 0.05.*

## Discussion

In plants, some small biological molecules, such as phytohormones and signal molecules could be considered as a powerful tool in modifying plants’ adaptability against adverse environment (Mostofa et al., 2015a). By exogenously application of the H_2_S donor NaHS, numerous results demonstrated that H_2_S participates in plant adaptive responses against multiple abiotic stresses (Lai et al., 2014). Ameliorative effects of H_2_S treatment are a trending topic among researchers that work on abiotic stress in plants, accordingly there are various studies in the literature that investigate how this molecule increase plant stress tolerance. However, most of the studies are limited to a certain topic such as only photosynthesis or Na^+^ and K^+^ homeostasis or antioxidant defense. A comprehensive study that investigates several issues in the same experimental setup and provides evidence on how H_2_S alleviates detrimental effects of salt stress is still limited. Our hydroponically based study aids in understanding how plants respond under NaCl-toxicity and how the use of H_2_S is capable of reverting this toxicity.

### H_2_S Alleviates NaCl-Induced Inhibition of Plant Growth

Results showed that salinity stress inhibited plant growth performance as judged by decreased shoot fresh weight, root fresh weight, shoot dry weight, root dry weight, leaf area and root length compared with control, but this inhibition was significantly alleviated by H_2_S donor NaHS (Figure 1). Similarly, the reversion of salt stress responses by H_2_S has been observed in other plant species, like *Medicago sativa* (Lai et al., 2014), *Oryza sativa* (Mostofa et al., 2015b) and *Triticum aestivum* (Deng et al., 2016). However, it is well-known that H_2_S must be within a rather narrow concentration range to act as a signal molecule and that high concentrations of H_2_S induce a toxicity response. In our study, the capacity of H_2_S to enhance salt tolerance firstly increased and then decreased with the concentration improvement. Ameliorative effects of phenotypes were seen from 5 to 15 μM NaHS, but plants exhibited slight toxicity under the 20 μM NaHS treatment. It may be that excessive high levels of H_2_S might lead to the ROS over-production, which further leads to the inhibition of the mitochondrial electron transport chain (Mancardi et al., 2009). In our study, 20 μM NaHS treatment indeed caused the ROS over-production and cell membrane injury than 15 μM NaHS as judged by the parameters of electrolyte leakage, MDA, O_2_^•-^ and H_2_O_2_ (Figure 3). Hence, the use of H_2_S is a double-edged sword, and the maintenance of a suitable level of intracellular H_2_S is key conferring salt tolerance in cucumber seedlings.

### H_2_S Alleviates the NaCl-Induced Declines of Photosynthetic Attributes

In this study, the F_v_/F_m_ was significantly decreased under salt stress, and they were remarkably improved with addition of 15 μM NaHS (Table 1). Similarly, the results of Christou et al. (2013) showed that 100 mM NaCl stress significantly decreased the values of F_v_/F_m_, while 100 μM NaHS significantly reversed the decline of F_v_/F_m_. In addition, a phenomenon that H_2_S prevented the reduction of photosynthetic pigments C_a_, C_b_, C_a+b_, and C_x+c_ induced by salt stress was also observed in our study (Table 1). The results are consistent with these of Mostofa et al. (2015b), who reported that H_2_S improved overall growth and biomass of salt-stressed rice plants which could be attributed to its role in protecting Chl_a_, Chl_b_ and Cx+c from salt-induced damage. As the photosynthetic parameters P_n_ and T_r_, our results showed that H_2_S also alleviated the decrease in the photosynthetic parameters P_n_ and T_r_. These results are consistent with these of Dawood et al. (2012), who reported that H_2_S reduced the Aluminum-induced inhibition of P_n_, G_s_ and T_r_. Another study also demonstrated a similar result that NaHS treatment increased the P_n_ and G_s_ values in *Spinacia oleracea* seedlings under drought (Chen et al., 2016). Together these results suggest that H_2_S plays a role in plant photosynthesis.

### Exogenous H_2_S Changes Endogenous H_2_S Accumulation Under Excess NaCl

Earlier investigations demonstrated that hypoxia, salt and Cd stress induce notable increases in endogenous H_2_S production in different plant species, such as pea, strawberry, alfalfa and *Chinese cabbage* (Cheng et al., 2013; Christou et al., 2013; Lai et al., 2014; Zhang et al., 2015). The activation of endogenous H_2_S level after stress treatments indicated that H_2_S might also be an important secondary messenger of stress sensing which in turn modulated plant physiological changes and downstream gene expressions (Christou et al., 2013). Surprisingly, a remarkable decline in endogenous H_2_S production was observed in cucumber leaves and roots upon treatment with 200 mM NaCl for 7 days in our experiment. Further investigation is still required to detail the reason. Although different kinds of environmental stress factors caused differential dynamic changes in endogenous H_2_S metabolism, exogenously applied NaHS, a well-known H_2_S donor, all greatly enhanced H_2_S concentration.

Generally, synthesize H_2_S in plants is associated with the enzymes L-CD, D-CD, OAS-TL or CAS, often in response to environmental stress, leading to accumulation of endogenous H_2_S and the acquisition of stress tolerance. The activities of L-CD in leaves and D-CD in both roots and leaves all showed a significantly decreased after salt stress treatment as compared to the control (Figure 4C–F). Lai et al. (2014) found that NaCl could induce L-CD in *Medicago sativa* roots, while the total activity of D-CD was not significantly altered. Meanwhile the expression of L-CD and D-CD at the transcripts level were strongly induced by drought stress in *Arabidopsis thaliana* (Jin et al., 2011). In our study, exogenously applied 15 μM NaHS significantly enhanced the activities of D-CD in both roots and leaves and L-CD in roots compared with the control or salt stress group (Figure 4D–F). However, Cheng et al. (2013) found that exogenously applied NaHS decreased the total activity of L-CD and D-CD in *Pisum sativum* L. seedlings under hypoxia stress. In addition, NO could induce the activities of to L-CD, OAS-TL and CAS regulate the endogenous H_2_S biosynthesis in maize roots (Peng et al., 2016). In our study, salt-stress significantly improved the activity of CAS in leaves and OAS-TL in leaves and roots and significantly reduced the activity of CAS in roots (Figure 4G–J). This is also in line with a report of Cheng et al. (2013), and their results showed that exogenously applied NaHS enhanced the activity of CAS and OAS-TL in *Pisum sativum* L. seedlings under hypoxia stress (Cheng et al., 2013).

### H_2_S Maintains Na^+^ and K^+^ Homeostasis by Regulating Expression of *PM H^+^-ATPase*, *SOS1*, and *SKOR* Genes Under Excess NaCl

Maintenance of Na^+^ and K^+^ ion homeostasis is critically important for plants to survive under salt stress. Our study provided firm evidence that H_2_S prevented Na^+^ uptake and improved K^+^ uptake in both leaves and roots, thereby restraining the salt-induced decrease of the Na^+^/K^+^ ratio (Figure 5). Similarly, Lai et al. (2014) observed that the biologically beneficial role of NaHS was due to its specific ability to retain K^+^, thus preventing the increase of the Na^+^/K^+^ ratio in stellar cells under salt stress. In addition, Christou et al. (2013) showed that H_2_S regulates the expression of SOS pathway genes and kept the balance of Na^+^ and K^+^ in strawberry plants under salt stress. Deng et al. (2016) also found that H_2_S significantly improved the salt tolerance of wheat seedlings by regulating the membrane-bound translocation proteins of the *SOS1* pathways to increase Na^+^ extrusion and decrease Na^+^ uptake. Interestingly, salt stress induced the up-regulation of *PM H^+^-ATPase*, *SOS1* and *SKOR* at the transcript levels in roots compared with the control, but a completely opposite trend in leaves. This is also in line with a report of Wang et al. (2013), and their results showed that the expression of *CsSOS1* increased in roots and decreased in leaves with the concentration of NaCl increasing in cucumber. Perhaps the root irrigation treatment method in this study affected the expression of genes or some signal first in roots. In our study, H_2_S alleviated the NaCl-induced increases in *PM H^+^-ATPase* expression in roots. It has been known that NaCl stress also up-regulates the expression of genes encoding *PM H^+^-ATPase* in roots (Shi et al., 2000). Moreover, it is well known that *PM H^+^-ATPase* can sustain an H^+^ gradient by promoting Na*^+^* efflux and H*^+^* influx to drive Na^+^/H^+^ antiport across the plasma membrane. It was also found that the administration of NaHS effectively prevented the NaCl-triggered K^+^ efflux in the mature zone of alfalfa seedling roots, which might be partially related to the *SKOR* channel (Lai et al., 2014). These studies suggested that salt-tolerant need plants have an ability to prevent transport of Na^+^ from roots to shoots and an improved ability to exclude Na^+^ from the roots (Takahashi et al., 2007a,b).

### H_2_S Regulates the Antioxidant System and Reduces Oxidative Stress and Membrane Lipid Peroxidation Under Excess NaCl

Plants suffering from NaCl toxicity often exhibit symptoms associated with oxidative stress and membrane lipid peroxidation, which can result in accumulation of ROS and MDA (Hossain et al., 2005). In this study, high salinity triggered overproduction of O_2_^•-^ and H_2_O_2_, increased the electrolyte leakage, and caused accumulation of MDA in both cucumber leaves and roots (Figure 3). H_2_S alleviated the salinity-induced oxidative damage, as evident by reduced levels of O_2_^•-^, H_2_O_2_, MDA and electrolyte leakage in both cucumber leaves and roots (Figure 3). This is in line with a report of Mostofa et al. (2015b), and their results showed that H_2_S reduced the salt-induced increase in the levels of O_2_^•-^, H_2_O_2_ and MDA in rice. Similar results were also reported by Chen et al. (2016), who demonstrated that NaHS treatment counteracted the accumulation of ROS and increased the MDA content of *Spinacia oleracea* L. seedlings under drought. The balance between ROS production and antioxidant defense determines the extent of oxidative damage within the plant. In our study, NaHS treatment significantly alleviated the improvement of ASA and GSH contents and SOD, GR, and POD activities in leaves of cucumber under salt stress (Table 2). In cucumber roots, H_2_S also successfully counteracted the decline in the content of ASA and GSH and the activities of CAT, GR and POD under salt stress (Table 2), indicating that H_2_S may enhance the ability of cucumber roots to scavenge ROS so that the plant can maintain membrane integrity under salt stress. The similar result was reported by Wang et al. (2012), who founded that NaHS pretreatment significantly activated the antioxidant enzymes activities including SOD, CAT, POD, and APX and their expression at the transcripts level under 100 mM NaCl stress in *Medicago sativa* L., thus resulting in the alleviation of oxidative damage induced by NaCl.

## Conclusion

**FIGURE 7:**
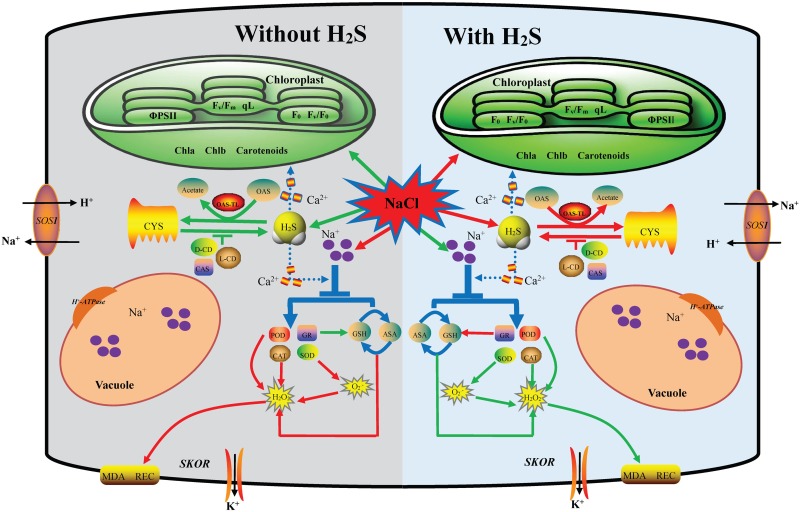
A proposed model for H_2_S-mediated salt stress responses in cucumber. This model depicting how H_2_S regulates the enhanced salt tolerance by mediating maintaining the Na^+^/K^+^ balance and regulating endogenous H_2_S metabolism and oxidative stress response. Under salt stress, on the one hand, the application of NaHS reversed the expression up-regulation of *PM H^+^-ATPase*, *SOS1*, and *SKOR* in roots to keep Na^+^ and K^+^ balance. On the other hand, the application of NaHS increase L/D-CD activity and decrease CAS and OAS-TL activities to regulating endogenous H_2_S metabolism. Additionally H_2_S altered the accumulation of antioxidants and the activities of antioxidant enzymes, leading to reduced ROS accumulation and membrane lipid peroxidation. The red arrow indicated up-regulation or increase and the green arrow indicated down-regulation or decrease in this model.

In conclusion, exogenous application of H_2_S alleviates the NaCl-induced toxicity via improving the growth and photosynthetic parameters in cucumber, which could be attributed to three mechanisms: (1) H_2_S increases endogenous H_2_S levels via increasing the activities of L-CD and D-CD and decreasing the activities of CAS and OAS-TL in leaves or roots; (2) H_2_S maintains the Na^+^ and K^+^ balance through regulating the expression of *PM H^+^-ATPase, SOS1* and *SKOR*; and (3) H_2_S enhances the accumulation of antioxidants and the activities of antioxidant enzymes, leading to decline ROS accumulation and membrane lipid peroxidation. As summarized in Figure 7, H_2_S alleviates salt stress in cucumber through cross talk among the mechanisms that maintain Na^+^/K^+^ homeostasis and regulating H_2_S metabolism and oxidative stress response. In addition, the roots and leaves of cucumber showed different responses to exogenous H_2_S treatment. Taken together, suitable concentration H_2_S could be used to alleviate NaCl-stress induced toxicity in cucumber seedlings.

## Author Contributions

J-LJ designed the experiment and wrote the manuscript. YT and X-MR conducted the experiment. MY and R-PH helped in data analysis and presentation. LL revised the manuscript. All authors listed have made a substantial, direct and intellectual contribution to the work.

## Conflict of Interest Statement

The authors declare that the research was conducted in the absence of any commercial or financial relationships that could be construed as a potential conflict of interest.

## Supplementary Material

Figure S1 Effects of NaHS treatment on the morphological changes **(A)**, survival rate **(B)**, and expanding rate of the second leaf **(C)** in seedlings of *Cucumis sativus* L. cv. Chunxiaqiuwang grown under 200 mM NaCl. Each value is the mean of three biological replicates, and the vertical bars represent the standard errors. Values sharing the same lower case letters are insignificant as per Duncan’s test at *P* < 0.05.

## Abbreviations

Φ_PSII_: Quantum yield of photosystem II photochemistry
^1^O_2_: Singlet oxygen
ANOVA: One-way analysis of variance
APX: Ascorbate peroxidase
ASA: Ascorbic acid
CAS: Cyanoalanine synthase
CAT: Catalase
CO: Carbon monoxide
Cx+c: Carotenoid
D-CD: D-cysteine desulfhydrase
DCIP: 2,6-dichlorophenolindophenol
DTT: DL-dithiothreitol
EC1: Initial electrical conductivity
EC2: Second electrical conductivity
EDTA: Ethylenediaminetetraacetic acid
ETR: Electron transport rate
F_m_: Maximal chlorophyll fluorescence
F_m′_: Maximum fluorescence level in the light-adapted state
F_o_: Minimal chlorophyll fluorescence
F_o′_: Minimal fluorescence level in the light-adapted state
F_v_/F_o_: Ratio of variable to minimal fluorescence
F_v_/F_m_: Ratio of variable to maximum fluorescence
FW: Fresh weight
GPX: Glutathione peroxidase
GR: Glutathione reductase
GSH: Glutathione
H^+^-ATPase: ATP-dependent proton pump
H_2_O_2_: Hydrogen peroxide
H_2_S: Hydrogen sulfide
K^+^: Potassium ions
L-CD: L-cysteine desulfhydrase
MDA: Malondialdehyde
Na^+^: Sodium ions
NADPH: Nicotinamide adenine dinucleotide phosphate
NaHS: Sodium hydrosulfide
NO: Nitric oxide
NPQ: Non-photochemical quenching parameters
NSCCs: Non-selective cation channels
O_2_^•-^: Superoxide radical anions
OAS-TL: O-acetyl-(thiol)-serinelyase
OH: Hydroxyl radicals
PAR: Photosynthetically active radiation
PM H^+^-ATPase: ATP-dependent proton pump of the plasma membrane
P_n_: Net photosynthetic rate
POD: Peroxidase
PSII: Photosystem II
QA: primary electron acceptor of PSII
qP: Photochemical quenching
qRT-PCR: Quantificational real-time polymerase chain reaction
ROS: Reactive oxygen species
RT-PCR: Real-time polymerase chain reaction
SKOR: Shaker-like K^+^ outward-rectifying channel
SOD: Superoxide dismutase
SOS: Salt overly sensitive
TBA: Thiobarbituric acid
TCA: Trichloroacetic acid
Tr: Transpiration rate
