# Supplementary figures and images for: H2S Alleviates Salinity Stress in Cucumber by Maintaining the Na+/K+ Balance and Regulating H2S Metabolism and Oxidative Stress Response
Source: Front Plant Sci. 2019 May 28;10:678. doi: 10.3389/fpls.2019.00678 (PMC6555442; doi:10.3389/fpls.2019.00678)

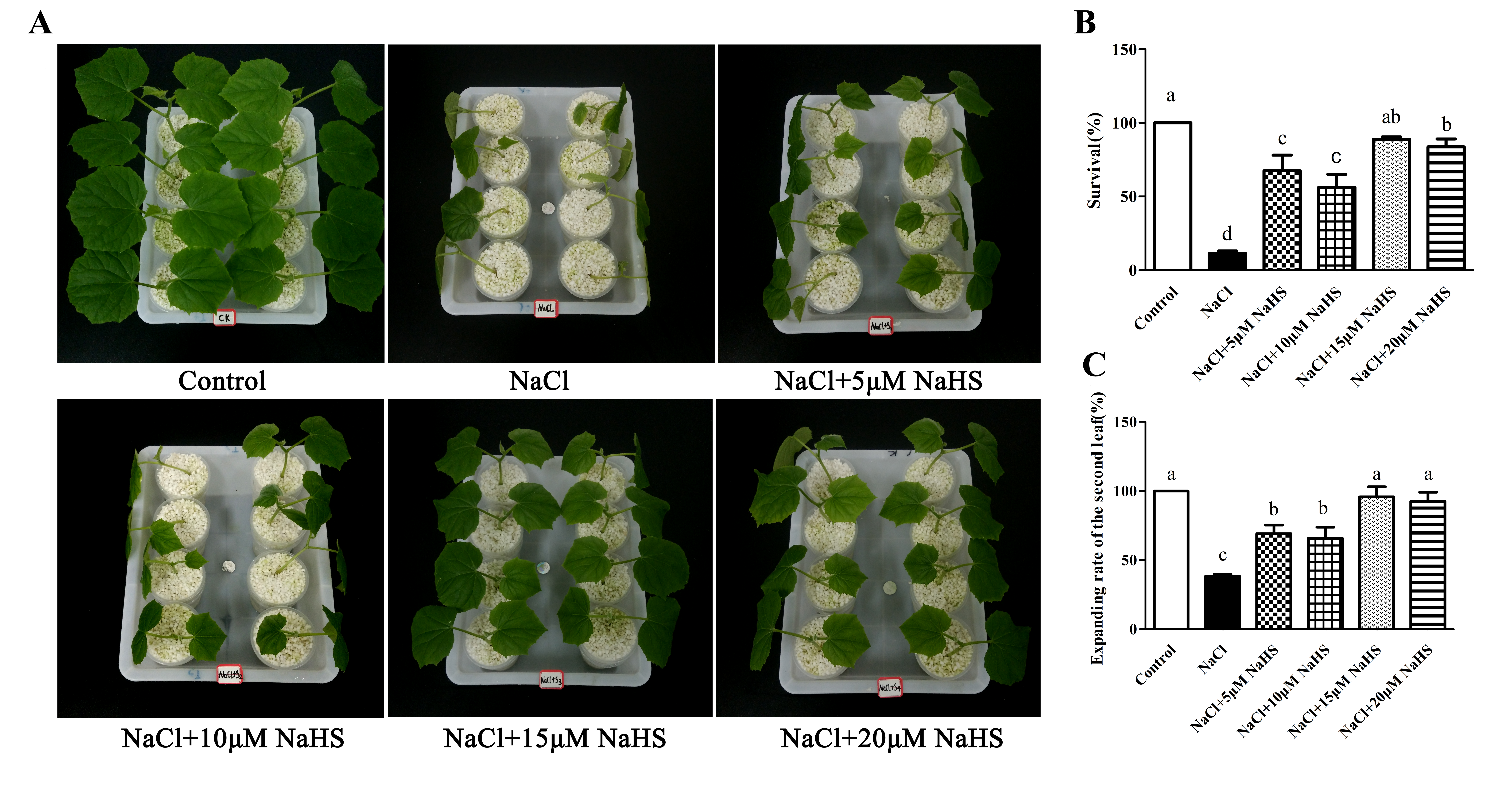

Supplement: Figure S1 — Effects of NaHS treatment on the morphological changes (A), survival rate (B), and expanding rate of the second leaf (C) in seedlings of Cucumis sativus L. cv. Chunxiaqiuwang grown under 200 mM NaCl. Each value is the mean of three biological replicates, and the vertical bars represent the standard errors. Values sharing the same lower case letters are insignificant as per Duncan’s test at P < 0.05. [file Image_1.TIF]
