# Supplementary material for: H2S Alleviates Salinity Stress in Cucumber by Maintaining the Na+/K+ Balance and Regulating H2S Metabolism and Oxidative Stress Response
Source: Front Plant Sci. 2019 May 28;10:678. doi: 10.3389/fpls.2019.00678 (PMC6555442; doi:10.3389/fpls.2019.00678)
Supplement: Supplementary file 2 [file Table_1.DOC]

**Table S1 |** Names and sequences of oligonucleotide primers used for qRT-PCR amplification.

| Gene | Primer | Sequence (5’-3’) |
| --- | --- | --- |
| *SOS1* | Zep-F | TGCGGAACCATCTCATAGCC |
|  | Zep-R | CTCACCACCCGATTCCTCAC |
| *SKOR* | Zep-F | GATTATGACGGGAGGTCGCC |
|  | Zep-R | GAATCGCCCCTTGAAACTGC |
| *H*^+^*-ATPase* | Zep-F | TACCGTGACGGAATCGACAA |
|  | Zep-R | CAATGGCAGTCATACGCTTC |
| *GAPDH* | Zep-F | CCTACCGTTGATGTCTCTGTTGTT |
|  | Zep-R | TTCCCTCGGACTCTTCCTTG |
